# Feasibility of Minimally Invasive Surgery After Chemo-Immunotherapy in Locally Advanced and Oligometastatic NSCLC: Technical Aspects and Surgical Outcomes

**DOI:** 10.3390/cancers18142244

**Published:** 2026-07-13

**Authors:** Giorgio Cannone, Luigi Lione, Viola Sambataro, Alessandro Bonis, Vincenzo Verzeletti, Alessandro Rebusso, Giovanni Maria Comacchio, Eleonora Faccioli, Giulia Pasello, Laura Bonanno, Fiorella Calabrese, Samuele Nicotra, Marco Schiavon, Andrea Dell’Amore

**Affiliations:** 1 Unit of Thoracic Surgery, Department of Cardiac, Thoracic, Vascular Sciences and Public Health, University of Padova, Via Giustiniani, 2, 35128 Padua, Italy; giorgio.cannone@aopd.veneto.it (G.C.); viola.sambataro@aopd.veneto.it (V.S.); alessandro.bonis@aopd.veneto.it (A.B.); vincenzo.verzeletti@aopd.veneto.it (V.V.); alessandro.rebusso@aopd.veneto.it (A.R.); giovannimaria.comacchio@unipd.it (G.M.C.); eleonora.faccioli@unipd.it (E.F.); marco.schiavon@unipd.it (M.S.); andrea.dellamore@unipd.it (A.D.); 2 Department of Surgery, Oncology and Gastroenterology, University of Padova, 35128 Padua, Italy; giulia.pasello@unipd.it (G.P.); laura.bonanno@unipd.it (L.B.); 3 Pathological Anatomy, Department of Cardiac, Thoracic, Vascular Sciences and Public Health—DCTV, University of Padova Medical School, 35121 Padova, Italy; fiorella.calabrese@unipd.it; 4 Unit of Thoracic Surgery, S. Orsola-Malpighi Hospital, Via Massarenti 9, Pavillion 23rd, 40138 Bologna, Italy; samuele.nicotra@aosp.bo.it

**Keywords:** non-small cell lung cancer (NSCLC), neoadjuvant immunotherapy, minimally invasive thoracic surgery

## Abstract

**Background**: In patients with non-small cell lung cancer (NSCLC) treated with neoadjuvant chemo-immunotherapy, pulmonary resection can be technically demanding because of hilar/mediastinal inflammation and fibrosis, potentially limiting the use of minimally invasive surgery (MIS). We report our single-center experience with MIS anatomical lung resections after neoadjuvant chemo-immunotherapy. **Methods**: We retrospectively collected consecutive NSCLC patients who underwent neoadjuvant chemo-immunotherapy followed by anatomical lung resection with an intended MIS approach at our institution between May 2018 and October 2025. Primary endpoints were feasibility and safety (conversion rate, intraoperative and postoperative complications, need for reoperation, and 30-day mortality). **Results**: Fifty-two patients were included. Mean age was 65.9 ± 8.6 years; 27 (51.9%) were male. Median Charlson Comorbidity Index was 3 (IQR 2–4). Most patients had clinical stage III disease (IIIA 40.4%, IIIB 30.8%). Conversion to open surgery occurred in 7 cases (13.5%). One intraoperative complication was recorded (1.9%). Anatomical resections were predominantly lobectomies (including combined/extended lobectomies) (88.4%); sleeve resections accounted for 11.5%, and bilobectomy for 7.7%. Postoperative complications occurred in 13 patients (25.0%); reoperation for complications was required in 1 case (1.9%). No deaths were recorded in the dataset. Pathological response showed pCR in 22 patients (42.3%) and MPR in 10 (19.2%). **Conclusions**: In our experience, minimally invasive anatomical lung resection after neoadjuvant chemo-immunotherapy for NSCLC is feasible, with an acceptable conversion rate and manageable morbidity, and is associated with a substantial rate of pathological response. Careful patient selection and surgical expertise remain essential.

## Simple Summary

Immunotherapy combined with chemotherapy is increasingly used before surgery in patients with non-small cell lung cancer, as it can shrink tumors and improve pathological response. However, these treatments may also make surgery more difficult by causing inflammation and fibrosis around the lung vessels, bronchi, and lymph nodes. In this single-center study, we evaluated 52 patients who underwent planned minimally invasive lung resection after neoadjuvant chemo-immunotherapy. We found that this approach was feasible and safe in carefully selected patients, with a conversion rate to open surgery of 13.5%, postoperative complications in 25.0% of patients, and no deaths within 30 days. The main reason for conversion was fibrosis around vascular or bronchial structures. Our findings suggest that minimally invasive surgery after chemo-immunotherapy can be safely performed in experienced centers, provided that surgeons carefully plan the procedure and convert to open surgery when needed for safety.

## 1. Introduction

Lung cancer remains a leading cause of cancer-related mortality worldwide, and non-small cell lung cancer (NSCLC) accounts for the majority of cases. Surgical resection is the standard of care for early-stage disease. In contrast, locally advanced NSCLC has historically required multimodal treatment because of the high risk of locoregional persistence and distant recurrence [1,2,3,4]. In recent years, neoadjuvant and perioperative chemo-immunotherapy have substantially changed this treatment paradigm. The addition of immune checkpoint inhibitors (ICIs) to platinum-based chemotherapy has increased major pathological response (MPR) and pathological complete response (pCR) rates, while improving patients’ survival compared with standard chemotherapy alone [5,6].

**Figure 1:**
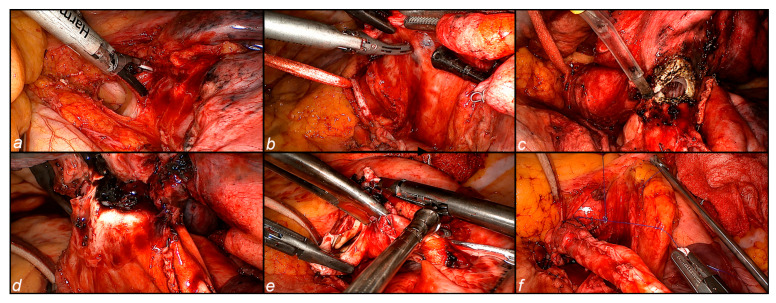
**Key technical steps of vascular and bronchial management.** (**a**) Opening of the pericardium to expose the intrapericardial vascular structures; (**b**) isolation of the main pulmonary artery to obtain proximal vascular control; (**c**) bronchial transection; (**d**) temporary control of the main pulmonary artery using a tourniquet; (**e**) controlled transection of the pulmonary artery; (**f**) vascular suturing/reconstruction.

From a surgical perspective, the potential oncological benefits of neoadjuvant chemo-immunotherapy must be balanced against treatment-related operative complexity. Despite these remarkable oncological advances, surgery after chemo-immunotherapy has introduced new technical challenges. Dense hilar fibrosis, inflammatory adhesions, obliteration of tissue planes, and vascular fragility frequently complicate minimally invasive dissection and may require intraoperative adaptation of surgical strategy (Figure 1).

These changes may raise concerns regarding the safety, reproducibility, and feasibility of minimally invasive surgery (MIS) in this setting [6,7,8,9,10,11]. Although several studies have demonstrated that minimally invasive surgery is feasible after neoadjuvant chemo-immunotherapy, relatively few have specifically focused on the intraoperative technical challenges, practical surgical strategies, and decision-making processes required to safely complete these procedures.

We report our single-center experience with intended minimally invasive anatomical lung resection after neoadjuvant chemo-immunotherapy in patients with NSCLC, focusing on surgical feasibility, perioperative safety, technical complexity, and early oncological outcomes.

## 2. Materials and Methods

### 2.1. Patients and Variables

We retrospectively analyzed patients with locally advanced NSCLC and selected patients with oligometastatic stage IVA NSCLC who were surgically treated with radical intent after chemo-immunotherapy at our center between May 2018 and October 2025. Only patients whose procedures were initially planned through a minimally invasive approach via video-assisted thoracic surgery (VATS) were included. Patients who underwent intentional open surgery were excluded.

Collected anonymized variables included demographic and clinical characteristics, baseline staging, treatment details, operative data, postoperative outcomes, and histopathological findings. Baseline work-up included histological diagnosis, clinical staging according to the eighth edition of the TNM classification, PET/CT when available, and pulmonary function testing. Mediastinal staging was performed according to European Society of Thoracic Surgeons guidelines [12].

All the patients were discussed on a multidisciplinary tumor board (MTB) and received the best systemic treatment based on PD-L1 levels and clinical stage. All patients considered eligible for immunotherapy underwent comprehensive molecular profiling by next-generation sequencing before treatment initiation. No actionable driver alterations were detected, and all patients were wild type for the analyzed genes. In the oligometastatic group, only NSCLC with a single extrathoracic metastasis in a single organ was included [13].

The primary endpoints were feasibility and safety of the intended minimally invasive approach. These were assessed through conversion rate to thoracotomy and reason for conversion, duration of surgery, intraoperative and postoperative complications, need for reoperation, length of hospital stay, chest tube duration, and 30-day mortality. Secondary endpoints included type of pathological response, radical resection rate, relapse pattern, and early survival status. Among intraoperative complications, minor bleeding refers to low-pressure, controllable bleeding from small blood vessels (generally under 3–4 mm) like segmental or sub-segmental arterial branches. Conversely, major bleeding was defined as a rupture of a larger pulmonary vessel, such as the lobar or main pulmonary artery branch.

Conversion to thoracotomy was not considered a technical failure. It was defined as a safety endpoint and an accepted component of the operative strategy when exposure, oncological radicality, or secure vascular and bronchial control could not be ensured through MIS.

### 2.2. Patient Selection for Minimally Invasive Surgery

Following completion of neoadjuvant treatment, all patients underwent multidisciplinary re-evaluation, including contrast-enhanced CT, PET/CT scan, and bronchoscopy when a lobar bronchial involvement was suspected. Patients were considered suitable for an intended minimally invasive approach when imaging demonstrated technical feasibility of complete resection without obvious invasion of the main pulmonary artery, main bronchus, carina, or trachea. Radiological response was considered during surgical planning but was not regarded as an absolute prerequisite for MIS.

### 2.3. Operative Technique

Operative reports were reviewed to identify treatment-related intraoperative challenges. Particular attention was paid to lymph node adherence to the pulmonary artery or bronchus, perivascular and peribronchial fibrosis, fused fissures, and the reason for conversion when it occurred.

**Figure 2:**
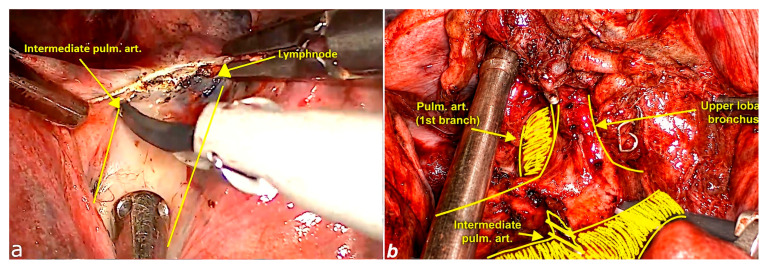
**Treatment-related fibrosis and bronchovascular dissection.** (**a**) Lymphonodal adhesions and fibrotic tissue involving the vascular plane; (**b**) detailed view after dissection.

Several operative principles were applied to reduce intraoperative risk. Dissection was performed in a stepwise anatomical fashion, with early identification of major vascular structures whenever feasible. Sharp dissection was preferred over forceful blunt dissection in fibrotic planes, particularly around the pulmonary artery and bronchus. In cases of difficult dissection and/or challenging identification of distal vascular structures, a proximal vascular control was carried out, and the main pulmonary artery was isolated and encircled, even intrapericardially when required (Figure 2). This technique is, in our opinion, a very safe tool, especially in cases of intraoperative rupture of arterial branches. In this setting, temporary closure of the main PA allows controlled management of vascular injury and facilitates vascular repair, while avoiding excessive blood loss: our first goal remains patient safety. In selected cases in which fibrosis made the interbronchovascular or broncho-arterial plane indistinguishable, a controlled sharp bronchial transection with a scalpel was used to expose the adjacent pulmonary arterial branch, allowing safe control and closure of the artery. When an adequate bronchial stump remained, bronchial closure was then completed, either with a stapler or by direct suture, according to the residual anatomy and the surgeon’s judgment. In patients with fused fissures or inflammatory fissural fibrosis, a fissure-last strategy was used when appropriate. Bronchoplastic and broncho-vascular procedures were performed when required to preserve lung parenchyma and avoid pneumonectomy while maintaining oncological radicality.

### 2.4. Pathological Evaluation

Pathological response was assessed according to the International Association for the Study of Lung Cancer (IASLC) guidelines, and the percentage of viable tumor cells, necrosis, and stroma was determined. A Major Pathological Response (MPR) was defined as the presence of no more than 10% of viable tumor cells in the primary tumor bed [14,15], while a Complete Pathological Response (pCR) was defined as the absence of viable tumor cells in the tumor bed. Non-Response (NR) occurred in all the other cases (viable tumor cells > 10%).

### 2.5. Statistical Analysis

Statistical analyses were performed using jamovi software, version 2.3.28 (the jamovi project, Sydney, Australia). Continuous variables were reported as mean ± standard deviation or median with interquartile range, as appropriate, while categorical variables were reported as frequencies and percentages.

## 3. Results

During the study period, a total of 65 patients were surgically treated with radical intent at our institution. Among them, 13 patients were excluded because surgery was planned through an upfront open thoracotomy approach; therefore, 52 patients were included in the study. The general characteristics of the patients are shown in Table 1. Mean age at diagnosis was 65.9 ± 8.6 years; 27 patients (51.9%) were male, and 25 (48.1%) were female. Most patients were former smokers (37, 71.2%), with a median smoking exposure of 40 pack-years (IQR 25–54). Median Charlson Comorbidity Index was 3 (IQR 2–4). Adenocarcinoma was the most frequent histology (35 patients, 67.3%), followed by squamous-cell carcinoma (16 patients, 30.8%).

At baseline, most patients were clinically staged IIIA and IIIB in 21 (40.4%) and 16 patients (30.8%). Pre-treatment pathological hilar lymph node involvement (N1) was detected in 28 patients (53%); among them, 21 also had concurrent N2 metastases, while isolated N2 involvement was observed in 14 cases. Nine patients were at stage IVA disease (17.3%), with a single extrathoracic metastasis. The metastatic sites were the central nervous system (CNS) in 4 patients, the bone in 3 patients, and the adrenal gland in 2 patients. All metastatic sites received locoregional definitive treatment: radiotherapy in 3 patients, surgery in 5 patients, and surgery combined with radiotherapy in 1 patient. These treatments allowed a local control of the metastatic site with no residual disease.

All patients received preoperative chemo-immunotherapy. Patients with oligometastatic stage IV disease were treated according to the established standard therapy protocols and with a locoregional curative-intent treatment as previously described. All the metastatic sites achieved complete local eradication before lung tumor surgery. The median interval between the end of neoadjuvant treatment and surgery was 37 days (IQR 31–51).

The median number of chemo-immunotherapy cycles administered was 4 (IQR, 4–4), with a median treatment duration of 9.5 weeks (IQR, 9.0–10.7). All procedures were planned with MIS intent (Table 2). Conversion to thoracotomy was required in 7 patients (13.5%). The most common reason for conversion was the presence of perivascular or peribronchial fibrosis (5 patients, 71.4%), due to treatment-related inflammatory effects, which made safe surgical dissection impossible. One conversion was related to documented first arterial PA branch infiltration, and one to minor bleeding. One intraoperative complication was recorded (1.9%), consisting of minor bleeding from a small PA branch during lymphadenectomy that was managed minimally invasively; no intraoperative death occurred. The main pulmonary artery was encircled in 12 cases, and in 7, a temporary clamping was required. The most frequent type of lung resection performed was lobectomy in 41 cases (78%). Among the lobectomy group, 6 cases included a bronchial and vascular double sleeve resection, and 2 cases an only-bronchial sleeve resection. Moreover, a chest wall resection was required in 2 patients due to chest wall infiltration. No pneumonectomies were performed.

We reported a radical resection in 51/52 patients (98%), since in one patient, fibrosis and extensive, scattered scar tissue surrounding the hilum and fissure made surgery impossible, even after conversion. Because of this, we carried out a wide wedge resection and tumor debulking in this instance in order to recharacterize the disease.

Median surgical time was 140 min (IQR 100–180), and median postoperative chest tube duration was 2.0 days (IQR 1.0–4.0) with a median length of hospital stay of 3.5 days (IQR 3.0–5.0).

Postoperative complications occurred in 13 patients, accounting for 16 postoperative events overall (Table 3). The most frequent specific events were prolonged air leaks; three of these patients received a conservative approach with bloodpatch pleurodesis, leading to a successful resolution of the condition. Conversely, one patient was surgically treated; this patient represented the only case requiring reoperation for postoperative complications (1.9%). No deaths occurred within 30 days.

Pathological complete response (pCR) was recorded in 22 patients (42.3%), MPR in 10 patients (19.2%), and non-response in 20 patients (38.5%). Median follow-up from surgery was 6.4 months (IQR 2.0–17.8). Relapse was recorded in 5 patients (9.6%), including locoregional relapse in 3 patients and distant relapse in 2 patients. At the time of the last follow-up, no deaths had been reported.

The main characteristics of the non-responder and responder groups are summarized in Table 4.

## 4. Discussion

This single-center experience suggests that intended minimally invasive lung resection after chemo-immunotherapy for NSCLC is feasible in carefully selected patients. In our practice, selection for MIS was based on a systematic and careful review of both pre-treatment and post-treatment CT scans. Patients were considered suitable for an intended MIS approach when imaging did not show clear invasion of the main hilar structures, such as the main pulmonary artery or main bronchus, or tumor extension too proximal to major vessels, the carina, or the trachea. Conversely, patients with radiological evidence of unresectable or highly complex central involvement requiring upfront open surgery were not selected for MIS. Hilar lymph node involvement (N1 disease) was not considered a contraindication to MIS, as reflected by the high proportion of patients with N1 disease in our cohort (53%). This subpopulation required sleeve resections for bronchial and/or arterial involvement by lymphadenopathies in 8 cases. Within this selected population, which nevertheless included a high proportion of patients with clinical stage III disease, conversion occurred in 7 patients (13.5%). However, none of the conversions occurred in an emergency instance. In fact, they were mainly due to intraoperative finding of scar tissue and fibrosis with no possibility of discriminating between treatment effects and presence of viable tumor (Figure 2). Therefore, conversion was necessary to guarantee oncological radicality. Moreover, we reported only one intraoperative complication: minor bleeding from a small segmental arterial branch, which was managed minimally invasively.

Thus, our findings support the safety of an intended minimally invasive strategy when performed by an experienced thoracic surgical team. The postoperative complication rate was 25%, the reoperation rate was 1.9%, and no 30-day mortality was recorded.

The main surgical message of this series is that feasibility should not be interpreted only as avoidance of thoracotomy. After chemo-immunotherapy, operative difficulty is frequently related to tissue quality and treatment-related effects rather than tumor size alone. Hilar and mediastinal inflammation, perivascular and peribronchial fibrosis, fused fissures, and lymphonodal tissue adherent to the pulmonary artery or bronchus may compromise safe dissection [7,8,11]. In our cohort, as previously described, perivascular and peribronchial fibrosis was the most common recorded reason for conversion. This finding supports the concept that conversion should be viewed as part of a safe, minimally invasive strategy rather than as a failure of the approach.

The broader oncological context is important [16,17,18]. Consolidation durvalumab after definitive chemoradiotherapy established immune checkpoint inhibition as a standard component of treatment for unresectable stage III NSCLC and provided part of the rationale for evaluating immunotherapy-based strategies in earlier and resectable disease settings [19].

Early neoadjuvant immunotherapy-alone studies, including the pilot experience with nivolumab and the LCMC3 trial with atezolizumab, demonstrated the biological activity of immune checkpoint blockade before surgery in resectable NSCLC [6,20]. However, these studies did not include concomitant platinum-based chemotherapy and therefore should not be directly compared with chemo-immunotherapy or perioperative chemo-immunotherapy cohorts in terms of pathological response, immune microenvironment remodeling, inflammatory changes, or surgical fibrosis.

Neoadjuvant chemo-immunotherapy trials, such as CheckMate 816 and NADIM, are more directly related to the surgical setting because they evaluated the impact of combined systemic treatment administered before resection [21,22]. CheckMate 816 demonstrated that neoadjuvant nivolumab plus platinum-based chemotherapy improved pathological complete response and event-free survival compared with chemotherapy alone, while subsequent survival data further supported the durability of this approach [21,23]. Similarly, NADIM showed encouraging pathological and clinical outcomes with neoadjuvant chemotherapy plus nivolumab in locally advanced resectable NSCLC [22]. Nevertheless, even in this setting, the exact contribution of immune activation, chemotherapy-induced necrosis, tumor regression, nodal sterilization, and residual viable disease to intraoperative fibrosis remains difficult to determine.

Perioperative trials, including AEGEAN, CheckMate 77T, and KEYNOTE-671, should be considered separately because they evaluate broader strategies combining neoadjuvant chemo-immunotherapy with postoperative adjuvant immunotherapy [24,25,26,27]. This distinction is particularly relevant to our cohort, in which most patients were treated according to perioperative treatment concepts. However, the operative findings reported in the present study specifically reflect the surgical impact of the preoperative component of these regimens, since resection was performed after neoadjuvant chemo-immunotherapy and before completion of the adjuvant phase.

Our relatively low conversion rate reflects the appropriate selection of the cases approached minimally invasively. In a recent meta-analysis, Bardoni et al. reported a pooled conversion-to-thoracotomy rate of 16.49% after neoadjuvant chemo-immunotherapy for NSCLC, with substantial heterogeneity across studies, likely reflecting differences in patient selection, disease stage, surgical approach, and surgeon expertise. Similarly, systematic reviews focused on MIS after neoadjuvant systemic therapy emphasize that minimally invasive resection can be safely performed in selected patients, provided that surgeons remain prepared to convert when vascular control, exposure, or oncological radicality cannot be safely guaranteed [28,29,30]. In this context, the 13.5% conversion rate observed in our VATS-dominant cohort enriched for clinical stage III disease appears acceptable and supports the feasibility of an intended MIS strategy when careful preoperative selection, early vascular control, and a low threshold for conversion are adopted. Recent evidence has suggested that robotic-assisted thoracic surgery (RATS) may be associated with lower conversion rates than conventional VATS following neoadjuvant chemo-immunotherapy, likely owing to enhanced three-dimensional visualization, improved instrument articulation, and greater dexterity during complex hilar dissection [31]. Nevertheless, direct comparisons remain limited and are mainly based on retrospective series with potential selection bias. In our institution, VATS remains the standard minimally invasive approach because of the surgical team’s extensive experience with this technique, from standard anatomical resections to more complicated surgeries with vascular and/or bronchial reconstructions. We believe that, particularly in the setting of post-induction surgery, surgeon expertise, meticulous patient selection, and readiness to convert when necessary are likely to have a greater impact on perioperative outcomes than the minimally invasive platform itself.

Several technical principles emerge from our experience. First, dissection should remain anatomical and stepwise, with early recognition of vascular structures and avoidance of uncontrolled traction on fibrotic nodal tissue. Second, in our experience, meticulous sharp dissection has often proved useful when dense fibrosis obscures normal tissue planes, particularly around the pulmonary artery and bronchus, as it may facilitate precise identification of anatomical structures while avoiding excessive traction on inflamed tissues. However, we acknowledge that no high-level evidence currently supports the superiority of sharp over blunt dissection in this setting, and the optimal technique should ultimately be tailored to the intraoperative anatomy and the surgeon’s experience. In cases of difficulty identifying the correct dissection plane on the pulmonary artery, our advice is to proximally control the main PA before persisting with difficult hilar dissection. This method may allow a rescue maneuver of PA closure in case of distal arterial branch injury. Temporary PA closure may be performed with a vascular clamp or tourniquet, depending on the surgeon’s preference.

Third, fissure-last dissection can be useful when the fissure is fused or inflamed. Finally, early conversion should be performed when safe exposure, vascular control, airway control, or oncological radicality cannot be guaranteed minimally invasively.

We acknowledge that these technical recommendations largely derive from institutional experience rather than high-level comparative evidence; nevertheless, they may provide practical guidance for thoracic surgeons increasingly confronted with post-immunotherapy surgical anatomy [32].

The pathological response rate in this cohort was substantial, with pCR observed in 42.3% of patients and MPR in 19.2%. These findings are consistent with the biological rationale for neoadjuvant chemo-immunotherapy. However, cross-study comparisons should be made cautiously because of differences in stage distribution and treatment regimens, and longer follow-up is required to determine whether these early pathological findings translate into durable oncological benefit.

The operative spectrum also deserves attention. This cohort included broncho-vascular sleeve and extended lobectomy resections. Moreover, sleeve lobectomy is now widely accepted as a safe procedure to achieve both radical resection of tumors invading the central structures with parenchymal sparing purpose.

These procedures indicate that MIS after neoadjuvant chemo-immunotherapy should not be restricted to simple lobectomies only. However, given their technical complexity and the potential need for advanced intraoperative decision-making and perioperative management, VATS sleeve lobectomies and other complex pulmonary resections should be performed in high-volume centers, where greater surgical expertise, multidisciplinary support, and higher procedural volumes are associated with improved patient outcomes and reduced perioperative morbidity. These findings also reinforce the need for careful patient selection, structured preoperative planning, advanced thoracic surgical expertise, and readiness to convert. In this setting, the goal is not MIS at all costs, but the safest oncologically sound resection.

This study has several limitations. It is retrospective, single-center, and includes a relatively small cohort with heterogeneous clinical stages. Because this was a descriptive single-arm cohort without an appropriate comparator group, propensity score matching or inverse probability weighting could not be applied.

Another limitation is the relatively long study period (2018–2025), during which the therapeutic landscape of resectable NSCLC evolved substantially with the progressive implementation of neoadjuvant and perioperative chemo-immunotherapy regimens. Consequently, some degree of treatment heterogeneity is unavoidable. Nevertheless, the inclusion of consecutive patients over this period reflects real-world clinical practice and allowed us to evaluate the evolution of minimally invasive surgical management following the introduction of these novel systemic therapies.

Follow-up remains short, limiting conclusions regarding disease-free and overall survival. Operative complexity was derived from operative reports rather than from a prospectively predefined surgical complexity score and therefore may be subject to reporting bias. Nevertheless, the study provides clinically useful real-world data on intended MIS after neoadjuvant chemo-immunotherapy and highlights practical determinants of intraoperative decision-making.

## 5. Conclusions

Neoadjuvant chemo-immunotherapy has reshaped the treatment paradigm of locally advanced and resectable NSCLC by increasing rates of major and complete pathological response and by expanding surgical candidacy within a multimodal strategy.

In this single-center experience, intended minimally invasive anatomical lung resection after chemo-immunotherapy was feasible and safe, with acceptable conversion rates, manageable morbidity, low reoperation rate, and no 30-day mortality. The most relevant determinant of conversion was perivascular or peribronchial fibrosis, underscoring that conversion should be interpreted as a safety measure rather than as failure of MIS.

Pulmonary resection after chemo-immunotherapy should be performed in experienced centers with careful preoperative planning, readiness for advanced reconstructive procedures, and a low threshold for conversion when vascular control, airway control, exposure, or oncological radicality are uncertain. Longer follow-up and prospective multicenter studies are warranted to validate these findings and clarify the relationship between pathological response, operative complexity, and long-term oncological outcomes.

## Figures and Tables

**Table 1:** Baseline Patient Characteristics.

Baseline Characteristics (*n* = 52)	
**Gender**	
Male	27 (51.9%)
Female	25 (48.1%)
**Age at diagnosis**	65.9 ± 8.6
**Smoking history**	
Never	3 (5.76%)
Smoker	12 (23.07%)
Former smoker	37 (71.2%)
**Pack/year**	40 (25, 54)
**PS ECOG**	
0	42 (80.8%)
1	10 (19.2%)
**Preoperative histology**	
Adenocarcinoma	35 (67.3%)
Squamous-cell carcinoma	16 (30.76%)
Carcinoma NOS	1 (1.9%)
**Clinical TNM stage VIII ed.**	
IIA	2 (3.8%)
IIB	4 (7.7%)
IIIA	21 (40.4%)
IIIB	16 (30.8%)
IVA	9 (17.3%)
**Type of ICIs**	
Nivolumab	22 (42.3%)
Atezolizumab	1 (1.9%)
Pembrolizumab	16 (30.7%)
Durvalumab	13 (25%)
**Post-operative Stage**	
ypT0N0	22 (42.3%)
ypTxN0	2 (3.8%)
ypT1aN0	4 (7.7%)
ypT2bN0	3 (5.8%)
ypT1aN1a	2 (3.8%)
ypT2N0	2 (3.8%)
ypTxN2	1 (1.9%)
ypTxN2a2	1 (1.9%)
ypT1N0	1 (1.9%)
ypT2aN1a	1 (1.9%)
ypT3N0	1 (1.9%)
ypT3N2	1 (1.9%)
ypT2N2a1	1 (1.9%)
ypT4N2b	1 (1.9%)
ypT2aN0	1 (1.9%)
ypT4N1a	1 (1.9%)
ypT2N1b	1 (1.9%)
ypT1bN0	1 (1.9%)
ypT1aN2	1 (1.9%)
ypT2bN1	1 (1.9%)
ypT1aN2a1	1 (1.9%)
ypT4N0	1 (1.9%)
ypT1bN2a2	1 (1.9%)
**Type of response**	
pCR	22 (42.31%)
MPR	10 (19.2%)
NR	20 (38.46%)

Abbreviations: pCR: Pathological Complete Response; MPR: Major Pathological Response; NR: Non Responder.

**Table 2:** Surgical characteristics.

Surgical Characteristics	
**Surgical approach: VATS**	52 (100%)
**Surgical resection**	
Lobectomy	31 (59.6%)
Segmentectomy	1 (1.9%)
Wedge resection	1 (1.9%)
Bilobectomy	4 (7.7%)
Sleeve	2 (3.8%)
Lobectomy and chest wall resection	2 (3.8%)
Lobectomy and segmentectomy	5 (9.6%)
Sleeve lobectomy with vascular reconstruction	6 (11.5%)
**Surgical time (min)**	140 (100, 180)
**Conversion to open surgery**	
No	45 (86.53%)
Yes	7 (13.46%)
**Reason for conversion**	
Minor Bleeding	1 (1.9%)
Perivascular or peribronchial fibrosis	5 (9.61%)
Vascular infiltration	1 (1.9%)
**Intraoperative complications**	
No	51 (98.07%)
Yes	1 (1.9%)
**Type of intraoperative complications**	
Minor bleeding	1

**Table 3:** Postoperative Complications.

Postoperative Complications	
No	39 (75%)
Yes	13 (25%)
**Type of complications**	
Empyema	1
Atrial fibrillation	3
Prolonged air leak	4
Other	8
**Necessity for reoperation**	
No	51 (98.07%)
Yes	1 (1.9%)

**Table 4:** Patient Characteristics.

Characteristic	N	NR *n* = 20 (38%)	R *n* = 32 (62%)	*p*-Value
**Age at diagnosis (y)**	52	66 (62, 74)	68 (60, 71)	0.85
**Operative time (min)**	52	165 (138, 204)	130 (88, 176)	0.009
**Conversion open surgery**	52			0.41
No		16 (80%)	29 (91%)	
Yes		4 (20%)	3 (9.4)	
**Intraoperative complications**	52			0.99
No		20 (100%)	31 (97%)	
Yes		0 (0%)	1 (3.1%)	
**Postoperative complications**	52			0.99
No		15 (75%)	24 (75%)	
Yes		5 (25%)	8 (25%)	

## Data Availability

The data presented in this study are available from the corresponding author upon reasonable request. The data are not publicly available due to privacy and ethical restrictions related to patient confidentiality.

## Abbreviations

The following abbreviations are used in this manuscript:

NSCLC	Non-Small Cell Lung Cancer
MIS	Minimally Invasive Surgery
VATS	Video-Assisted Thoracic Surgery
ICI	Immune Checkpoint Inhibitor
MPR	Major Pathological Response
pCR	Pathological Complete Response
NR	Non Responder
PA	Pulmonary Artery
MTB	Multidisciplinary Tumor Board

## References

[1] Alberg A.J., Brock M.V., Samet J.M. (2005). Epidemiology of Lung Cancer: Looking to the Future. J. Clin. Oncol..

[2] Crinò L., Weder W., van Meerbeeck J., Felip E., ESMO Guidelines Working Group (2010). Early Stage and Locally Advanced (Non-Metastatic) Non-Small-Cell Lung Cancer: ESMO Clinical Practice Guidelines for Diagnosis, Treatment and Follow-Up. Ann. Oncol..

[3] Nicholson A.G., Tsao M.S., Beasley M.B., Borczuk A.C., Brambilla E., Cooper W.A., Dacic S., Jain D., Kerr K.M., Lantuejoul S. (2022). The 2021 WHO Classification of Lung Tumors: Impact of Advances Since 2015. J. Thorac. Oncol..

[4] Rami-Porta R., Nishimura K.K., Giroux D.J., Detterbeck F., Cardillo G., Edwards J.G., Fong K.M., Giuliani M., Huang J., Kernstine K.H. (2024). The International Association for the Study of Lung Cancer Lung Cancer Staging Project: Proposals for Revision of the TNM Stage Groups in the Forthcoming (Ninth) Edition of the TNM Classification for Lung Cancer. J. Thorac. Oncol..

[5] Brunelli A., Charloux A., Bolliger C.T., Rocco G., Sculier J.-P., Varela G., Licker M., Ferguson M.K., Faivre-Finn C., Huber R.M. (2009). ERS/ESTS Clinical Guidelines on Fitness for Radical Therapy in Lung Cancer Patients (Surgery and Chemo-Radiotherapy). Eur. Respir. J..

[6] Forde P.M., Chaft J.E., Smith K.N., Anagnostou V., Cottrell T.R., Hellmann M.D., Zahurak M., Yang S.C., Jones D.R., Broderick S. (2018). Neoadjuvant PD-1 Blockade in Resectable Lung Cancer. N. Engl. J. Med..

[7] Bott M.J., Cools-Lartigue J., Tan K.S., Dycoco J., Bains M.S., Downey R.J., Huang J., Isbell J.M., Molena D., Park B.J. (2018). Safety and Feasibility of Lung Resection After Immunotherapy for Metastatic or Unresectable Tumors. Ann. Thorac. Surg..

[8] Mathey-Andrews C., McCarthy M., Potter A.L., Beqari J., Wightman S.C., Liou D., Raman V., Yang C.-F.J. (2022). Safety and Feasibility of Minimally Invasive Lobectomy after Neoadjuvant Immunotherapy for Non-Small Cell Lung Cancer. J. Thorac. Cardiovasc. Surg..

[9] Bott M.J., Yang S.C., Park B.J., Adusumilli P.S., Rusch V.W., Isbell J.M., Downey R.J., Brahmer J.R., Battafarano R., Bush E. (2019). Initial Results of Pulmonary Resection after Neoadjuvant Nivolumab in Patients with Resectable Non-Small Cell Lung Cancer. J. Thorac. Cardiovasc. Surg..

[10] Feldman H., Sepesi B., Leung C.H., Lin H., Weissferdt A., Pataer A., William W.N., Walsh G.L., Rice D.C., Roth J.A. (2023). Surgical Outcomes after Chemotherapy plus Nivolumab and Chemotherapy plus Nivolumab and Ipilimumab in Patients with Non-Small Cell Lung Cancer. J. Thorac. Cardiovasc. Surg..

[11] Comacchio G.M., Sewell M., Tan K.S., Toumbacaris N., Bott M.J., Adusumilli P.S., Chidi A., Gray K., Huang J., Isbell J.M. (2026). Feasibility and Oncologic Outcomes of Minimally Invasive Surgery after Induction Chemoimmunotherapy for Non-Small Cell Lung Cancer. JTCVS Open.

[12] De Leyn P., Dooms C., Kuzdzal J., Lardinois D., Passlick B., Rami-Porta R., Turna A., Van Schil P., Venuta F., Waller D. (2014). Revised ESTS Guidelines for Preoperative Mediastinal Lymph Node Staging for Non-Small-Cell Lung Cancer. Eur. J. Cardiothorac. Surg..

[13] Hellman S., Weichselbaum R.R. (1995). Oligometastases. J. Clin. Oncol..

[14] Travis W.D., Dacic S., Wistuba I., Sholl L., Adusumilli P., Bubendorf L., Bunn P., Cascone T., Chaft J., Chen G. (2020). IASLC Multidisciplinary Recommendations for Pathologic Assessment of Lung Cancer Resection Specimens After Neoadjuvant Therapy. J. Thorac. Oncol..

[15] Cottrell T.R., Thompson E.D., Forde P.M., Stein J.E., Duffield A.S., Anagnostou V., Rekhtman N., Anders R.A., Cuda J.D., Illei P.B. (2018). Pathologic Features of Response to Neoadjuvant Anti-PD-1 in Resected Non-Small-Cell Lung Carcinoma: A Proposal for Quantitative Immune-Related Pathologic Response Criteria (irPRC). Ann. Oncol..

[16] Yokota J., Shiraishi K., Kohno T. (2010). Genetic Basis for Susceptibility to Lung Cancer: Recent Progress and Future Directions. Adv. Cancer Res..

[17] Bardhan K., Anagnostou T., Boussiotis V.A. (2016). The PD1:PD-L1/2 Pathway from Discovery to Clinical Implementation. Front. Immunol..

[18] Verma S., Breadner D., Mittal A., Palma D.A., Nayak R., Raphael J., Vincent M. (2024). An Updated Review of Management of Resectable Stage III NSCLC in the Era of Neoadjuvant Immunotherapy. Cancers.

[19] Antonia S.J., Villegas A., Daniel D., Vicente D., Murakami S., Hui R., Yokoi T., Chiappori A., Lee K.H., De Wit M. (2017). Durvalumab after Chemoradiotherapy in Stage III Non-Small-Cell Lung Cancer. N. Engl. J. Med..

[20] Chaft J.E., Oezkan F., Kris M.G., Bunn P.A., Wistuba I.I., Kwiatkowski D.J., Owen D.H., Tang Y., Johnson B.E., Lee J.M. (2022). Neoadjuvant Atezolizumab for Resectable Non-Small Cell Lung Cancer: An Open-Label, Single-Arm Phase II Trial. Nat. Med..

[21] Forde P.M., Spicer J., Lu S., Provencio M., Mitsudomi T., Awad M.M., Felip E., Broderick S.R., Brahmer J.R., Swanson S.J. (2022). Neoadjuvant Nivolumab plus Chemotherapy in Resectable Lung Cancer. N. Engl. J. Med..

[22] Provencio M., Nadal E., Insa A., García-Campelo M.R., Casal-Rubio J., Dómine M., Majem M., Rodríguez-Abreu D., Martínez-Martí A., de Castro Carpeño J. (2020). Neoadjuvant Chemotherapy and Nivolumab in Resectable Non-Small-Cell Lung Cancer (NADIM): An Open-Label, Multicentre, Single-Arm, Phase 2 Trial. Lancet Oncol..

[23] Forde P.M., Spicer J.D., Provencio M., Mitsudomi T., Awad M.M., Wang C., Lu S., Felip E., Swanson S.J., Brahmer J.R. (2025). Overall Survival with Neoadjuvant Nivolumab plus Chemotherapy in Lung Cancer. N. Engl. J. Med..

[24] Heymach J.V., Harpole D., Mitsudomi T., Taube J.M., Galffy G., Hochmair M., Winder T., Zukov R., Garbaos G., Gao S. (2023). Perioperative Durvalumab for Resectable Non-Small-Cell Lung Cancer. N. Engl. J. Med..

[25] Cascone T., Awad M.M., Spicer J.D., He J., Lu S., Sepesi B., Tanaka F., Taube J.M., Cornelissen R., Havel L. (2024). Perioperative Nivolumab in Resectable Lung Cancer. N. Engl. J. Med..

[26] Spicer J.D., Garassino M.C., Wakelee H., Liberman M., Kato T., Tsuboi M., Lee S.-H., Chen K.-N., Dooms C., Majem M. (2024). Neoadjuvant Pembrolizumab plus Chemotherapy Followed by Adjuvant Pembrolizumab Compared with Neoadjuvant Chemotherapy Alone in Patients with Early-Stage Non-Small-Cell Lung Cancer (KEYNOTE-671): A Randomised, Double-Blind, Placebo-Controlled, Phase 3 Trial. Lancet.

[27] Garassino M.C., Torri V. (2024). Neoadjuvant or Perioperative Approach in Lung Cancer. N. Engl. J. Med..

[28] Bardoni C., Chiari M., Bertolaccini L., Diotti C., De Fabiani A., Nicolosi G., Mazzella A., Casiraghi M., Spaggiari L. (2025). Surgical Outcomes After Neoadjuvant Chemo-Immunotherapy for Stage III NSCLC: A Systematic Review and Meta-Analysis. Cancers.

[29] Trabalza Marinucci B., Mancini M., Siciliani A., Messa F., Piccioni G., D’Andrilli A., Maurizi G., Ciccone A.M., Menna C., Vanni C. (2025). Surgical Techniques for Non-Small-Cell Lung Cancer After Neoadjuvant Chemo-Immunotherapy: State of Art and Review of the Literature. Cancers.

[30] Zhang B., Xiao Q., Xiao H., Wu J., Yang D., Tang J., Li X., Wu Z., Zhou Y., Wang W. (2022). Perioperative Outcomes of Video-Assisted Thoracoscopic Surgery Versus Open Thoracotomy After Neoadjuvant Chemoimmunotherapy in Resectable NSCLC. Front. Oncol..

[31] Srivatsa S., Maréchal H., Altorki N.K., Villamizar N., Phillips J.D., Schnorr P., Jones D.R., D’Souza D., Baiu I., Abdel-Rasoul M. (2026). Multicenter Study Comparing Outcomes of Robotic Versus Video-Assisted Thoracoscopic Resection of Non-Small Cell Lung Cancer Following Neoadjuvant Chemoimmunotherapy. J. Robot. Surg..

[32] Schiavon M., Cannone G., Bertolaccini L., Gallina F.T., Pezzuto F., Lorenzoni G., Facciolo F., Spaggiari L., Calabrese F., Rea F. (2025). Safety and Efficacy of Salvage Surgery after Treatment with Immune-Checkpoint Inhibitors for Advanced Non-Small Cell Lung Cancer: A Multicentric Study. J. Surg. Oncol..

